# TGF-β Inducible Early Gene 1 Regulates Osteoclast Differentiation and Survival by Mediating the NFATc1, AKT, and MEK/ERK Signaling Pathways

**DOI:** 10.1371/journal.pone.0017522

**Published:** 2011-03-14

**Authors:** Muzaffer Cicek, Anne Vrabel, Catherine Sturchio, Larry Pederson, John R. Hawse, Malayannan Subramaniam, Thomas C. Spelsberg, Merry Jo Oursler

**Affiliations:** 1 Department of Biochemistry and Molecular Biology, Mayo Clinic College of Medicine, Rochester, Minnesota, United States of America; 2 Endocrine Research Unit, Mayo Clinic College of Medicine, Rochester, Minnesota, United States of America; Roswell Park Cancer Institute, United States of America

## Abstract

TGF-β Inducible Early Gene-1 (TIEG1) is a Krüppel-like transcription factor (KLF10) that was originally cloned from human osteoblasts as an early response gene to TGF-β treatment. As reported previously, TIEG1^−/−^ mice have decreased cortical bone thickness and vertebral bone volume and have increased spacing between the trabeculae in the femoral head relative to wildtype controls. Here, we have investigated the role of TIEG1 in osteoclasts to further determine their potential role in mediating this phenotype. We have found that TIEG1^−/−^ osteoclast precursors differentiated more slowly compared to wildtype precursors *in vitro* and high RANKL doses are able to overcome this defect. We also discovered that TIEG1^−/−^ precursors exhibit defective RANKL-induced phosphorylation and accumulation of NFATc1 and the NFATc1 target gene DC-STAMP. Higher RANKL concentrations reversed defective NFATc1 signaling and restored differentiation. After differentiation, wildtype osteoclasts underwent apoptosis more quickly than TIEG1^−/−^ osteoclasts. We observed increased AKT and MEK/ERK signaling pathway activation in TIEG1^−/−^ osteoclasts, consistent with the roles of these kinases in promoting osteoclast survival. Adenoviral delivery of TIEG1 (AdTIEG1) to TIEG1^−/−^ cells reversed the RANKL-induced NFATc1 signaling defect in TIEG1^−/−^ precursors and eliminated the differentiation and apoptosis defects. Suppression of TIEG1 with siRNA in wildtype cells reduced differentiation and NFATc1 activation. Together, these data provide evidence that TIEG1 controls osteoclast differentiation by reducing NFATc1 pathway activation and reduces osteoclast survival by suppressing AKT and MEK/ERK signaling.

## Introduction

During osteoclast formation, RANKL and M-CSF activate NFκB, c-Jun N-terminal kinase, ERK, and AKT [1], [2], [3], [4], [5], [6]. These signaling pathways also modulate osteoclast survival in response to RANKL and M-CSF. RANKL also activates or induces the expression of transcriptional factors important for osteoclastogenesis including c-Fos, MITF and NFATc1 [7], [8], [9]. NFATc1 is considered a master regulator of RANKL-induced osteoclastogenesis since reduced expression of NFATc1 causes defects in osteoclastogenesis in response to RANKL. NFATc1 is regulated by the serine/threonine phosphatase calcineurin, which is activated by intracellular Ca^2+^. Dephosphorylation of NFATc1 at serine residues by calcineurin stimulates NFATc1 to translocate into the nucleus [9]. A crucial gene target for NFATc1 in osteoclast precursors is dendritic cell-specific transmembrane protein (DC-STAMP), a “master fusion gene” for osteoclast differentiation [10], [11], [12]. Several other cellular components such as a disintegrin and metalloproteinase (ADAM) 8 and 12, adenosine A1 receptors, CD200 receptor, CD36, CD63, E-cadherin, filamin A, some integrins, some matrix metalloproteinases, a subunit of the v-ATPase, and the intracellular phosphatases SHP1 and 2 have also been implicated in regulating osteoclast fusion [13]. The mechanisms by which these diverse proteins function remain mostly unresolved [13].

TGF-β Inducible Early Gene-1 (TIEG1) was originally cloned from human osteoblasts as a primary response gene to TGF-β treatment [14]. TIEG1 is a member of the Krüppel-like transcription factor family (KLF10) which is expressed in numerous tissues [15], [16], [17], [18], [19] and is involved in the regulation of cell growth, differentiation and apoptosis [20], [21], [22]. We have previously demonstrated that TIEG1 knockout mice (TIEG1^−/−^) display a gender specific osteoporotic bone phenotype [23], [24]. Specifically, analysis of the distal femur metaphysis revealed a 44% decrease in cancellous bone volume (BV/TV) of congenic TIEG1^−/−^ mice compared to wildtype (WT) mice [24]. In this study, the number of osteoclasts in TIEG1^−/−^ mice remained unchanged from wildtype mice in spite of the defective ability of osteoblasts to support osteoclast differentiation [25]. Additionally, a recent study has demonstrated that TIEG1^−/−^ osteoblasts have increased expression of OPG suggesting that osteoclast differentiation in these animals could be impaired [26]. Because of this discrepancy, we examined osteoclast precursor differentiation in TIEG1^−/−^ bone marrow precursors. Here, we demonstrate that loss of TIEG1 reduces NFATc1 activation and slows the rate at which osteoclasts differentiate *in vitro*. Moreover, loss of TIEG1 in mature osteoclasts reduces apoptosis and results in increased activation of pro-survival AKT/NFκB and MEK/ERK signaling.

## Results

### Loss of TIEG1 Delays Osteoclast Differentiation *in Vitro*

**Figure 1:**
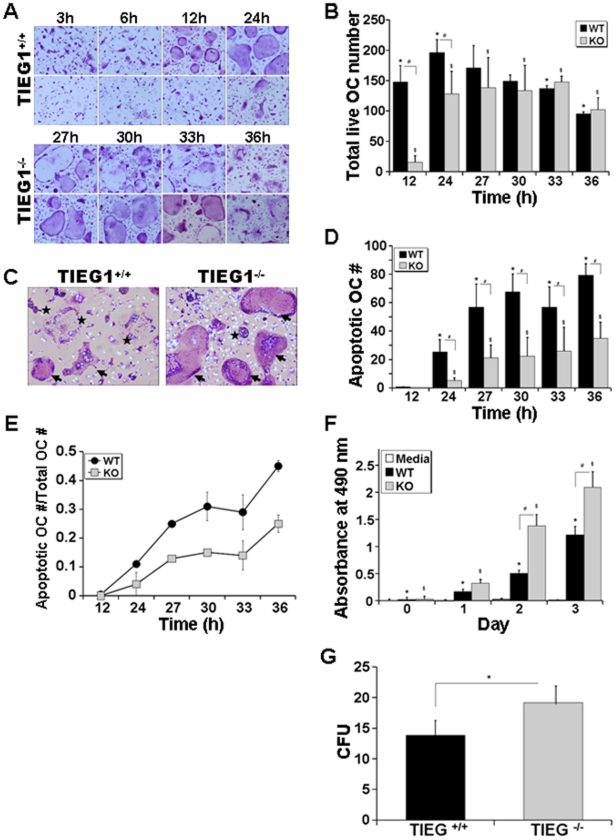
Lack of TIEG1 in osteoclast precursors delays osteoclast differentiation and apoptosis *in vitro*. **A**. WT and TIEG1^−/−^ marrow cells were cultured as described in the Methods section and subsequently fixed and stained for TRAP activity and chromatin condensation beginning on day 3 or after feeding the cells with MCSF and RANKL for the indicated time in hours (h). These data are representative of three separate experiments. **B**. Mean +/− SD of viable osteoclasts over time. These data were obtained from four replicate wells (p<0.05) and are representative of three separate experiments. **C**. Image of TRAP and Hoechst stained osteoclasts on day 4. Viable osteoclasts are indicated with an arrow and apoptotic osteoclasts are indicated with a star. These data are representative of three separate experiments. **D**. Mean +/− SD of the number of apoptotic osteoclasts on day 3 after feeding the cells with MCSF and RANKL for the indicated time in hours. Note that apoptosis is not observed until day 4 (24 h after feeding on day 3). **E**. The ratio of apoptotic osteoclasts to total number of osteoclasts is also presented as mean +/− SD. These data were obtained from four replicate wells. (p<0.05) and are representative of three separate experiments. **F**. Mean +/− SD of proliferation of WT and TIEG1^−/−^ osteoclast progenitors. WT and TIEG1^−/−^ non-adherent bone marrow cells were seeded at 1×10^5^ cells/well in 96-well plates and grown at 37°C for 3 h at day 0, 1, 2 and 3 of differentiation. Proliferation was determined using an absorbance of 490 nm and the CellTiter 96® AQueous One Solution Assay kit. These data were obtained from eight replicate wells (p<0.05) and are representative of three separate experiments. **G**. The mean +/− SD of number of colony forming units (CFU)-GM. These data are from three replicate wells (p<0.05) and are representative of three separate experiments.

We compared the ability of WT and TIEG1^−/−^ marrow-derived osteoclast precursors to differentiate *in vitro* into osteoclasts when treated with M-CSF and RANKL (Figure 1). The results of these studies revealed a significant delay in the ability of precursors from TIEG1^−/−^ marrow cells to differentiate compared to WT precursors (Figure. 1A and B). To ensure that there were no contaminating mesenchymal cells in our experiments which could contribute to the observed differences in osteoclast differentiation, we cultured the non-adherent cells from WT and TIEG1^−/−^ marrow in the absence of MCSF. As expected, none of these non-adherent cells survived in the absence of MCSF confirming that the non-adherent cells from both WT and TIEG1^−/−^ mice have minimal, if any, mesenchymal cell contamination (data not shown). During late stages of culture, the number of WT osteoclasts decreased due to apoptosis (Figure 1C, D and E), which is consistent with our previous report [27]. However, osteoclasts lacking TIEG exhibited significantly less apoptosis than WT osteoclasts. Because TIEG1 is known to suppress the proliferation rates of several cell types [16], [17], [28], we next investigated the impact of loss of TIEG1 expression on osteoclast precursor proliferation. The proliferation rate of TIEG1^−/−^ marrow cells was elevated in the presence of RANKL and MCSF compared to WT cells (Figure 1F). To examine the numbers of osteoclast precursors in the marrow, we analyzed the number of granulocyte/macrophage colony forming units (CFU-GMs) and found that there were significantly more colonies per well in the TIEG1^−/−^ marrow cultures relative to WT controls (Figure 1G).

### TIEG1 Suppresses Survival Signaling While Promoting NFATc1 Activation

**Figure 2:**
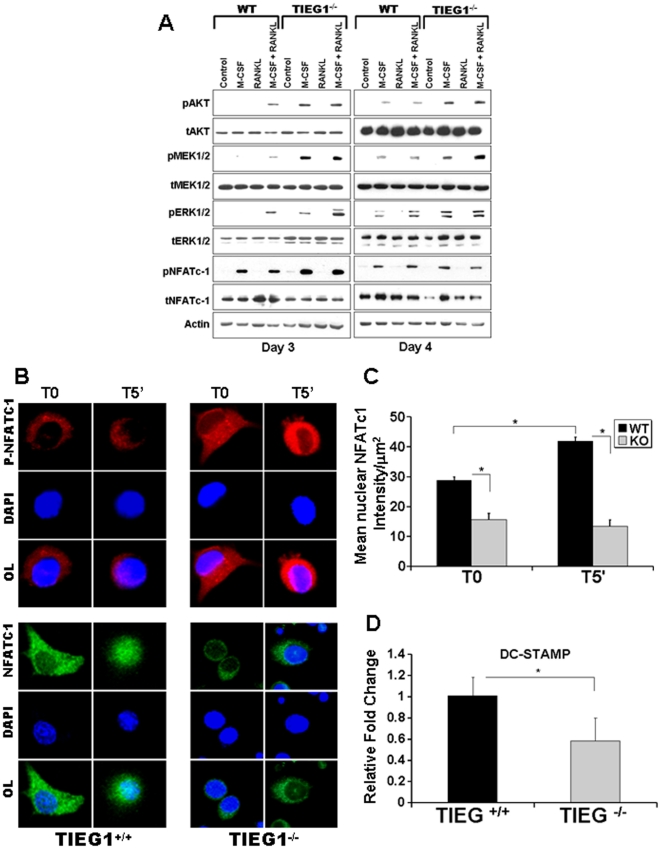
Signaling pathway analysis following MCSF and RANKL treatment. **A**. Osteoclast precursors at day 3 and mature osteoclasts at day 4 were serum-starved and treated with MCSF, RANKL or MCSF+RANKL as indicated for five minutes. Equal amounts of total protein were analyzed by western blotting for the indicated phospho (p) or total (t) proteins. These data are representative of two separate experiments. For each experiment, marrow cells from three mice were pooled and analyzed in three replicate wells. The data are presented as the mean +/− SD from all replicate wells. **B**. Confocal images of the effect of RANKL and MCSF treatment on phospho- NFATc1 and total NFATc1, respectively. These data are representative of two separate experiments. **C**. Quantitative nuclear NFATc1 was calculated at T0 and T5′ after RANKL and MCSF treatment. Precursors were cultured with MCSF and RANKL as in Figure 1. On day 3, the cells were rinsed and serum starved for 1 hour prior to ether fixing (T0) or 5 minutes (T5′) of treatment with M-CSF and RANKL. These data are representative of two separate experiments and each experiment is analyzed in three replicate wells. **D**. Loss of TIEG1 expression results in decreased DC-STAMP expression in pre-fusion day 3 precursors compared to WT day 3 precursors. These data are representative of two separate experiments. For each experiment, marrow cells from three mice were pooled and analyzed in three replicate wells. The data are presented as the mean +/− SD from all replicate wells.

AKT, MEK/ERK, and NFATc1 activation are required for osteoclast differentiation [6], [9], [29]. Moreover AKT and MEK/ERK activation are required for osteoclast survival [5], [30]. We compared activation of these pathways in osteoclast precursors and mature osteoclasts between WT and TIEG1^−/−^ marrow cells (Figure 2). We observed that M-CSF induced higher phosphorylation of AKT and MEK/ERK signaling in TIEG1^−/−^ precursors and mature osteoclasts compared to WT cells (Figure 2A). Phosphorylation of p38 and JNK did not differ between WT and TIEG1^−/−^ precursors and mature osteoclasts (data not shown). However, increased activation of MEK/ERK and AKT were not consistent with the reduced differentiation of TIEG1^−/−^ precursors. We therefore examined NFATc1 activation as this is the pivotal activation target of RANKL [9]. Unlike AKT, MEK, and ERK, phosphorylation of NFATc1 negatively influences activity as it is excluded from the nucleus when phosphorylated [9]. On day 3, TIEG1^−/−^ control precursors exhibited increased phosphorylation, thus decreased activation, of NFATc1 compared to WT cells (Figure 2A). Consistent with these data, we also observed similar activation of phospho-NFATc1, MEK/ERK and AKT in TIEG1^−/−^ precursors and mature osteoclasts from marrow derived cells from 16 month old mice (data not shown). To verify differential activation of NFATc1 between the two genotypes, we next examined osteoclast precursors for phospho- (Figure 2B) and total (Figure 2C) NFATc1 nuclear localization prior to and following 5 minutes of MCSF and RANKL treatment. As expected, these data demonstrated a decrease in phospho-NFATc1 nuclear localization in all cells (Figure 2B). However, TIEG1^−/−^ precursors exhibited increased cytoplasmic staining for phospho-NFATc1 regardless of M-CSF treatment compared to WT cells (Figure 2B). Quantitative examination of total NFATc1 staining revealed that, in WT precursors, the 5 minute MCSF and RANKL treatment led to increased nuclear localization whereas there was less evidence of nuclear localization in TIEG1^−/−^ osteoclast precursors (Figure 2C). We examined expression of genes that have been implicated in osteoclast fusion and found that loss of TIEG1 in pre-fusion day 3 precursors resulted in decreased DC-STAMP expression compared to WT day 3 precursors (Figure 2D). However, expression of other genes known to be involved in osteoclast fusion were not similarly suppressed in TIEG1^−/−^ day 3 precursors (Table S1).

### Effects on Differentiation Due to Loss of TIEG1 Expression are a Result of Defective RANKL Responses

**Figure 3:**
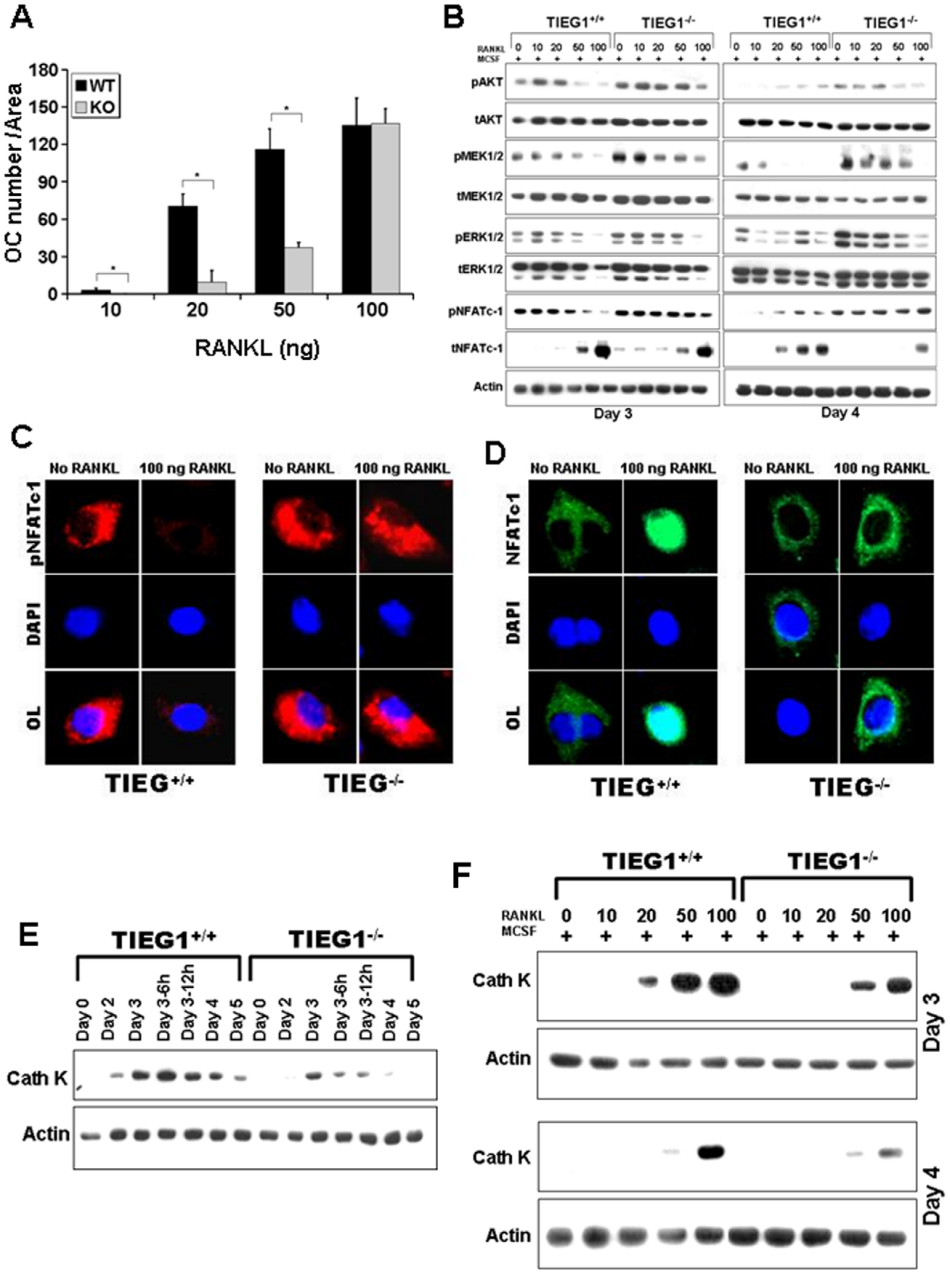
Differentiation defects in TIEG1^−/−^ osteoclast lineage cells results from defective RANKL responses. **A**. Mean number of osteoclasts from WT and TIEG1^−/−^ (KO) mouse marrow following 4 days of culture in the presence of 25 ng/ml MCSF and the indicated concentrations of RANKL as outlined in the Methods section. Cells were stained and the number of osteoclasts quantitated as in Figure 1 (*p<0.05). These data were obtained from three replicate wells (p<0.05) and are representative of three separate experiments. **B** Osteoclast precursors at day 3 and mature osteoclasts at day 4 were treated with the indicated concentration of RANKL and equal amounts of total protein were analyzed by western blotting for the indicated phospho- or total proteins. These data are representative of two separate experiments. For each experiment, marrow cells from three mice were pooled and analyzed in three replicate wells. **C and D**. Confocal images demonstrating the effect of RANKL on phospho- NFATc1 (**C**) or total NFATc1 (**D**). Precursors were cultured with MCSF with or without RANKL as indicated. On day 3, cells were fixed and stained with the indicated primary antibodies. These data are representative of two separate experiments and each experiment is analyzed in three replicate wells. **E and F**. Time course expression of cathepsin K in WT and TIEG1^−/−^ (KO) osteoclast precursors (day 3) and mature osteoclasts (day 4) cultured in the presence of MSCF alone (**E**) or with the indicated concentration of RANKL (**F**). Precursors and mature osteoclasts were harvested and cultured as in A for the indicated time. Equal protein from cell lysates were analyzed by western blotting for cathepsin K protein expression. These data are representative of two separate experiments. For each experiment, marrow cells from three mice were pooled and analyzed in three replicate wells.

The above observations suggested that altered RANKL signaling could be the cause of the delay in TIEG1^−/−^ osteoclast precursor differentiation. To evaluate this possibility, we tested a range of RANKL concentrations for their effects on differentiation. We observed that treatment with 100 ng/ml RANKL abolished the defect in osteoclast differentiation in TIEG1^−/−^ precursors (Figure 3A). Examination of RANKL effects on signaling pathway activation revealed that 100 ng/ml RANKL also reduced the differences between WT and TIEG1^−/−^ precursor signaling (Figure 3B). Specifically, we observed that dephosphorylation of NFATc1 was increased in WT precursors in the presence of 50 and 100 ng of RANKL when compared to TIEG1^−/−^ osteoclast precursors (Figure 3B). This observation was also confirmed by localization of phospho- (Figure 3C) and total (Figure 3D) NFATc1 which demonstrated that nuclear localization of NFATc1 was restored following 100 ng/ml RANKL treatment. These co-localization data revealed that increased phosphorylation of NFATc1 reduced nuclear localization of NFATc1 in osteoclasts precursors from TIEG^−/−^ mice in the presence of 100 ng RANKL (Figure 3B and C). Overall these observations support the hypothesis that increased phosphorylation of the NFATc1 pathway in TIEG1^−/−^ osteoclast precursors is likely to be the mechanism that leads to delayed osteoclast differentiation in the absence of TIEG1 expression. Since cathepsin K is a marker of osteoclast differentiation, we also examined the effect of loss of TIEG1 on cathepsin K expression during the differentiation time period (Figure 3E). We found that cathepsin-K expression was reduced in TIEG1^−/−^ osteoclast precursor cells when compared to WT osteoclast precursors in the presence of MCSF and RANKL (Figure 3E). In addition, we observed that WT cultures treated with low doses of RANKL exhibited higher levels of cathepsin K protein than TIEG1^−/−^ cultures and increased RANKL levels stimulated increased cathepsin K expression in late stages of differentiation of TIEG1^−/−^ precursors.

### AdTIEG1 expression in TIEG1^−/−^ OC precursors restores differentiation and signaling

**Figure 4:**
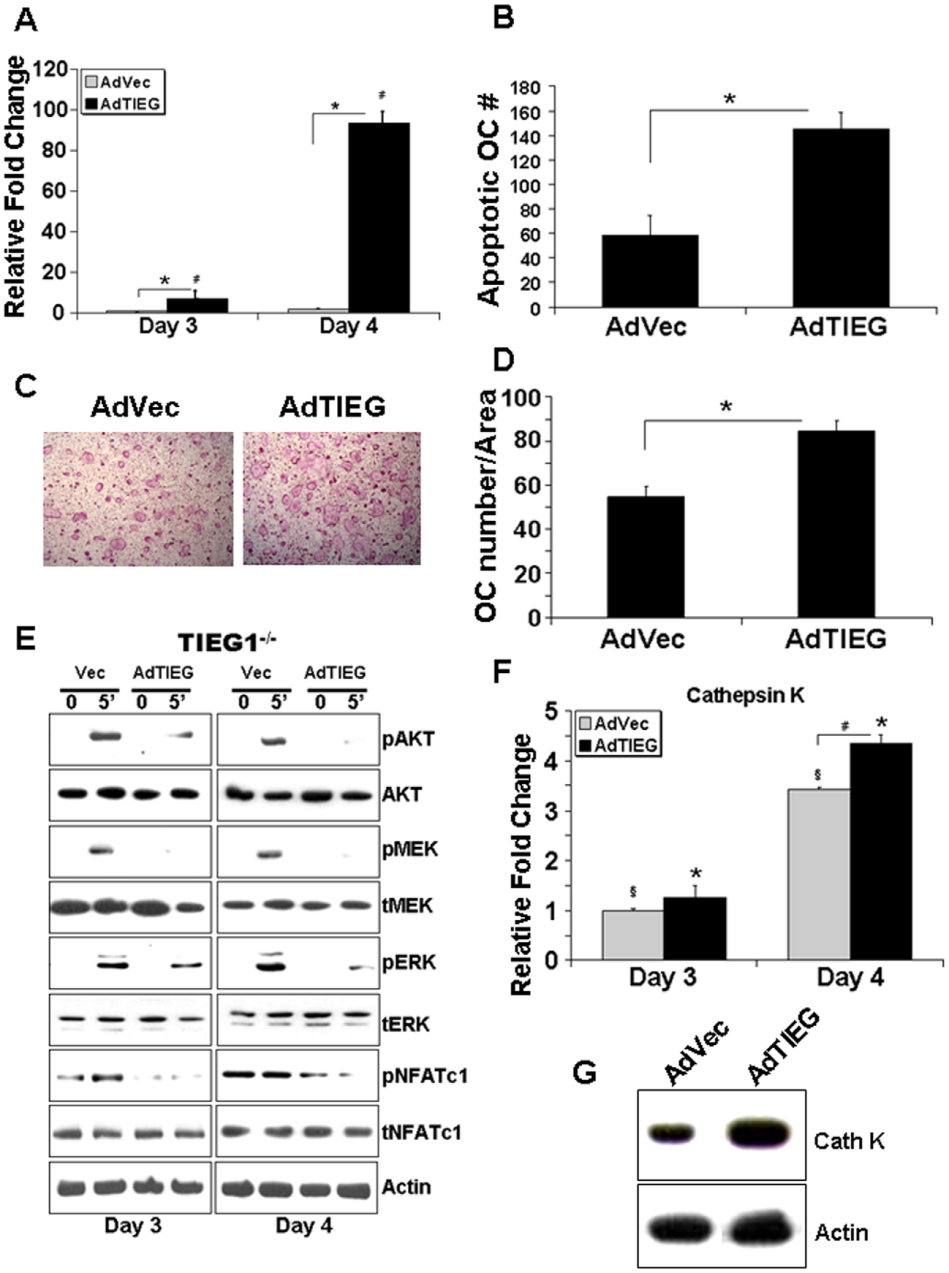
AdTIEG1 expression in TIEG1^−/−^ OC precursors restores differentiation and signaling defects. **A**. TIEG1^−/−^ precursor osteoclasts were infected at day 2 with vector control (AdVec) or TIEG1 (AdTIEG) adenovirus (MOI = 25) and TIEG expression was monitor by real time PCR at day 3 and day 4. These data were obtained from three replicate wells and are represented as the mean +/− SD (p<0.05). **B**. AdTIEG1 expression effects on osteoclast apoptosis. Osteoclast precursors were cultured as in A and apoptosis determined as in Figure 1D. Mean +/− SD of the number of apoptotic osteoclasts over time are depicted. These data were obtained from three replicate wells (p<0.05) and are representative of two separate experiments. Each experiment contained marrow cells pooled from three mice. **C and D**. AdTIEG1 expression effects on osteoclast differentiation. Osteoclast precursors were cultured as in A. **C** represents TRAP-stained vector and AdTIEG1-infected TIEG1^−/−^ osteoclasts at day 4 and **D** depicts the mean +/− SD of the number of osteoclasts quantitated. These data are from three replicate wells (p<0.05) and are representative of two separate experiments. Each experiment contained marrow cells pooled from three mice. **E**. AdTIEG1 expression effects on signaling. Osteoclast precursors were cultured as in A. Day 3 osteoclast precursors and mature osteoclasts at day 4 were serum-starved and either harvested or treated with MCSF and RANKL as indicated for five minutes. Equal amounts of total protein were analyzed by western blotting for the indicated phospho- or total proteins. These data are representative of two separate experiments. Each experiment contained marrow cells pooled from three mice. **F**. Expression of cathepsin K mRNA following restoration of TIEG1 expression. Cells were infected as in A and RNA was harvested. Samples were analyzed by Real Time PCR for cathepsin K. Data depict mean +/− SD and were obtained from three replicate wells (p<0.05) and are representative of three separate experiments. **G**. Expression of cathepsin K protein following restoration of TIEG1 expression. Cells were infected as in A and protein harvested 6 hours after feeding on day 3 and analyzed for cathepsin K expression levels by western blotting. These data are representative of two separate experiments. Each experiment contained marrow cells pooled from three mice.

We examined whether TIEG1 restoration *in vitro* in osteoclast precursors could restore the WT phenotype of TIEG1^−/−^ cells. We infected precursors from TIEG1^−/−^ mice with a mouse adenoviral TIEG1 (AdTIEG1) expression construct on day 2 at a multiplicity of infection (MOI) of 25. Controls were empty vector infected marrow cells from TIEG1^−/−^ mice. Expression of AdTIEG1 was determined by real time-PCR at days 3 and 4. Infection with AdTIEG1 significantly increased TIEG1 expression (Figure 4A). We next examined the influence of AdTIEG1 on mature osteoclasts apoptosis and differentiation of TIEG1^−/−^ precursors. We found that restoration of TIEG1 expression in TIEG1^−/−^ precursors resulted in increased apoptosis of mature cells (Figure 4B). Examination of differentiation influences revealed that adenoviral infected cultures differentiated ∼35% more when compared to vector infected cells (Figures 4C and 4D). We therefore infected precursors from TIEG1^−/−^ marrow as above and examined signaling responses. AdTIEG1 expression suppressed activation of the AKT and MEK/ERK survival pathways (Figure 4E). Consistent with the differentiation data, AdTIEG1 expression had a suppressive effect on the transient NFATc1 phosphorylation in TIEG1^−/−^ precursors. To confirm effects on differentiation, we examined cathepsin K expression responses. At both days 3 and 4, TIEG1 expression significantly increased mRNA expression of cathepsin K (Figure 4F). Cathepsin K protein levels were assessed 6 hours after feeding on Day 3 and increase protein expression was also observed following AdTIEG1 infection (Figure 4G).

### Suppression of TIEG1 inhibits differentiation and increases NFATc1 phosphorylation

**Figure 5:**
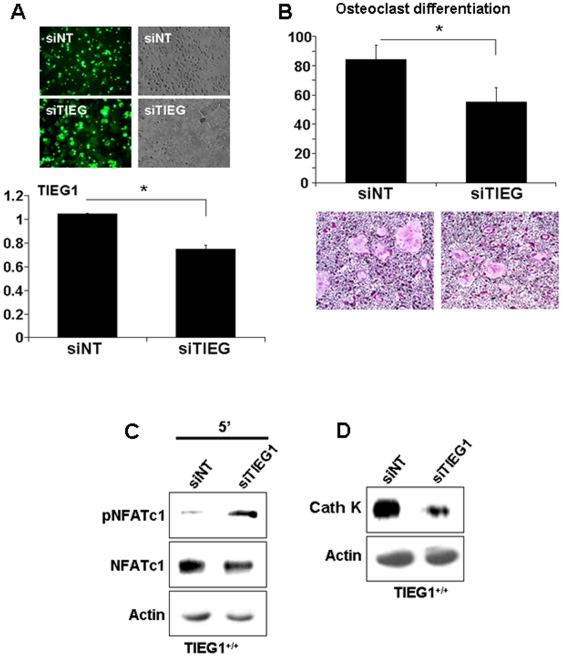
Blockade of TIEG1 expression in WT cells mimics TIEG1^−/−^ cell responses. Osteoclast precursors were transfected with accell-siNon-targetting (siNT) or accell-siTIEG1 as detailed in the Methods section. **A**. Wildtype osteoclast precursors were transfected at day 2 and TIEG1 expression was monitored by Real Time PCR on day 3. In the upper panel, green fluorescence demonstrates the transfection efficiency of accell-siNT and siTIEG1. The lower panel demonstrates the inhibition of TIEG1 expression by accell-siTIEG1 (mean +/− SD). These data were obtained from three replicate wells (p<0.05) and are representative of two separate experiments. Each experiment contained marrow cells pooled from three mice and analyzed in three replicate wells. **B**. siNT and siTIEG1 effects on osteoclast differentiation. WT osteoclast precursors were transfected with accell-siNT and siTIEG1 as in A and fixed and TRAP-stained on day 4. The upper panel is the quantitation of osteoclast number. The lower panel is a representative micrograph of TRAP-stained cultures. Data are presented as the mean +/− SD (p<0.05) and are representative of two separate experiments. Each experiment contained marrow cells pooled from three mice and analyzed in three replicate wells. **C**. siNT and siTIEG1 effects on NFATc1 phosphorylation. WT osteoclast precursors were treated as in A. Day 3 osteoclast precursors were serum-starved and either treated with MCSF or RANKL for five minutes. Equal amounts of total protein were analyzed by western blotting for phospho- or total NFATc1. **D**. siNT and siTIEG1 effects on cathepsin K expression. WT osteoclast precursors were treated as in A. Day 3 osteoclast precursor cell lystaes were harvested and equal amounts of total protein were analyzed by western blotting for cathepsin K expression. These data are representative of two separate experiments. Each experiment contained marrow cells pooled from three mice.

We used siRNA to block TIEG1 expression in WT cells to determine if we could recapitulate the TIEG1^−/−^ phenotype. Both non-targeting siRNA and siTIEG1 were fluorescently tagged to allow for an estimation of transfection efficiency. As shown in Figure 5A, siTIEG1 suppressed TIEG1 expression to about 26% relative to controls and the fluorescent tag indicated that a large portion (estimated at about 70%) of the cells contained the construct. Even this modest reduction in TIEG1 expression resulted in significant biological impacts and mimicked the TIEG1^−/−^ phenotype. Specifically, suppression of TIEG1 expression significantly decreased osteoclast differentiation of WT precursors (Figure 5B). Consistent with this, phosphorylation of NFATc1 was elevated in WT precursor cells containing siTIEG1 (Figure 5C) and cathepsin K protein expression was suppressed (Figure 5D).

### Effects of Loss of TIEG1 on Gene Expression

**Figure 6:**
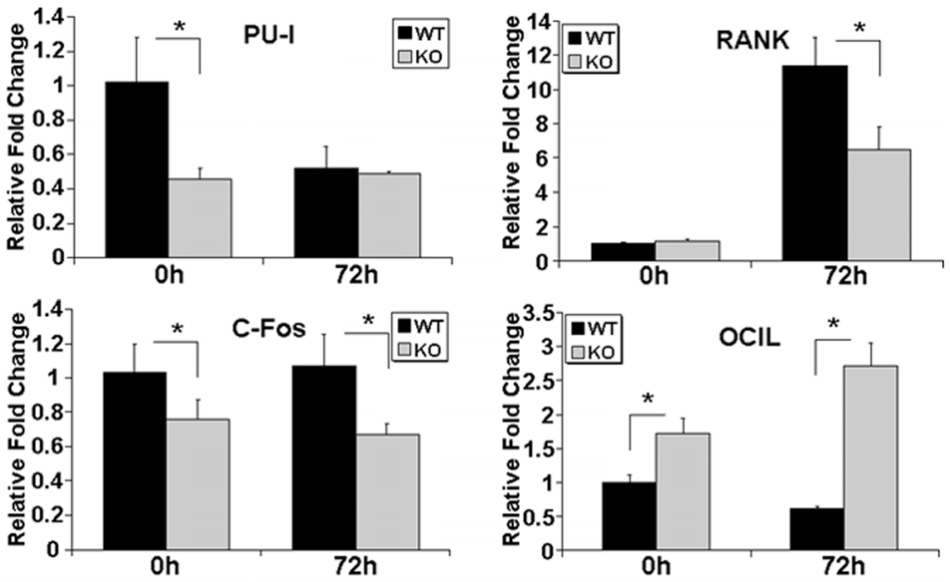
Effects of loss of TIEG1 expression on genes involved in osteoclast differentiation. Osteoclast precursors from WT and TIEG1^−/−^ (KO) mouse marrow were cultured as in Figure 1 for the indicated number of hours (h). Real Time PCR analysis of osteoclast differentiation marker genes, PU-1, RANK, c-fos, and OCIL was conducted at the indicated times. These data are presented as the mean +/− SD (p<0.05) and were obtained from four replicate wells. Data are representative of three separate experiments.

**Figure 7:**
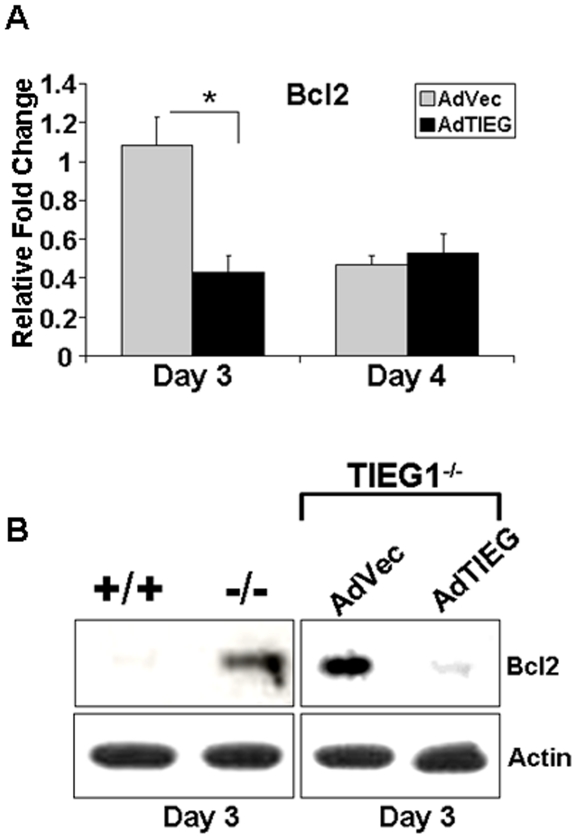
Effects of loss of TIEG1 expression on Bcl2 expression. **A**. TIEG1^−/−^ mouse marrow cells were cultured in the presence of MCSF and RANKL and infected at day 2 with vector or TIEG1 adenovirus. Bcl2 expression was monitor by real time PCR at day 3. These data were obtained from three replicate wells and are presented as the mean +/− SD (p<0.05). Data are representative of two separate experiments. Each experiment contained marrow cells pooled from three mice and analyzed in three replicate wells. **B**. Osteoclast precursors were cultured and infected at day 2 as in A. Day 3 osteoclast precursors were harvested and equal amounts of total protein were analyzed by western blotting for Bcl2 protein. These data are representative of two separate experiments. Each experiment contained marrow cells pooled from three mice.

We performed real time-PCR to analyze genes associated with osteoclastogenesis. We found a trend in that expression of c-fos, PU1, and RANK, the receptor for RANKL, were mostly down-regulated in TIEG1^−/−^ cells compared to WT controls (Figure 6). Expression of osteoclast inhibitory lectin (OCIL) was significantly up-regulated in TIEG1^−/−^ osteoclast lineage cells relative to those from WT mice (Figure 6). Evaluation of the effects of loss of TIEG1 on Bcl2 family members revealed no elevation of pro-survival members including Bcl2 and no suppression of pro-apoptosis family members (Table S2). Since we have previously shown that overexpression of Bcl2 can rescue mature osteoclast apoptosis [31], we examined the impact of AdTIEG infection on levels of Bcl2 gene and protein expression. We observed that late osteoclast TIEG1^−/−^ precursors expressed detectable levels of Bcl2 protein whereas WT precursors did not (Figure 7B). Infection of TIEG1^−/−^ precursors with AdTIEG suppressed Bcl2 mRNA and protein levels (Figure 7A and B).

## Discussion

To better understand the biological function of TIEG1 in osteoclastogenesis, we have investigated the role of TIEG1 in osteoclast precursor differentiation to determine if there is a defect that is independent of osteoblast influences. Osteoclast precursors isolated from female TIEG1^−/−^ mice differentiated more slowly and survived longer when compare to WT precursors. In bone, we have previously published that there are increased numbers of osteoblasts that exhibited an impaired ability to support osteoclast differentiation [25]. Our observation reported here is that there is likewise an impaired ability of osteoclast precursors lacking TIEG1 to fuse. These two observations would lead one to expect that there would be a reduced number of osteoclasts in TIEG1^−/−^ bones, which is not what was observed [23]. Longer culture of osteoclasts indicated that there was a reduction in apoptosis in TIEG1^−/−^ mature osteoclasts, allowing these cells to survive longer. This last observation suggests that the sustained number of osteoclasts in TIEG1^−/−^ bones is due to a reduction in osteoclast apoptosis. Reductions in apoptosis likely compensate for the impaired ability of TIEG1^−/−^ osteoblasts to support OC differentiation and reduced ability of TIEG1^−/−^ precursors to fuse and form multinucleated osteoclasts. We chose to examine early osteoclast precursors to determine whether the defect in differentiation was due to a reduction in the proliferative response to cytokines and/or due to fewer early progenitor cells. Loss of TIEG1 expression resulted in increased proliferation rates, eliminating that as the cause of the differentiation defect. The CFU assays indicate that there are significantly more early progenitors in the TIEG1^−/−^ marrow cultures also eliminating this as a cause of the defect as well. However, this later observation is consistent with the increased proliferation rate that we observed. The responses to restoration of TIEG1 in TIEG1^−/−^ cells and suppression of TIEG1 in WT cells confirm that the observed impacts of loss of TIEG1 are osteoclast lineage cell-autonomous defects and not simply the result of defects in osteoblast/osteoclast cross-talk.

**Figure 8:**
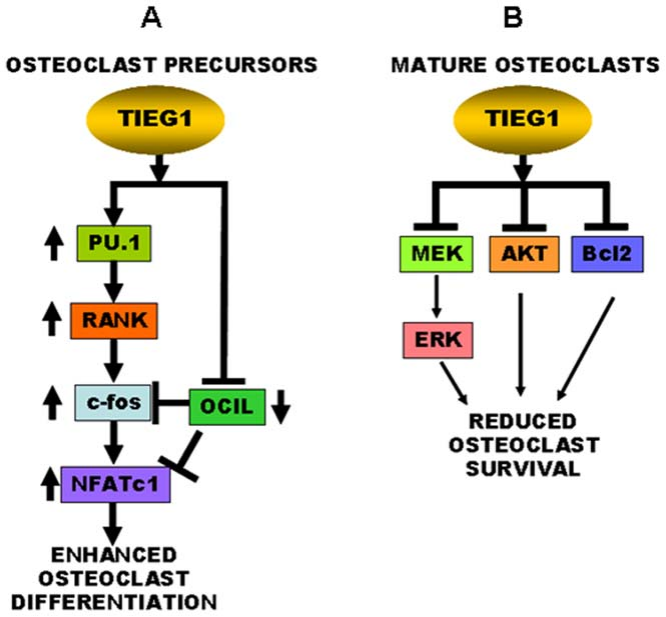
Proposed model for TIEG1 effects on osteoclast differentiation and survival. **A**. In osteoclast precursors, TIEG1 expression transiently increases expression of pro-differentiation PU.1, leading to increased RANK expression, which induces c-Fos expression. This, combined with suppression of the inhibitory osteoclast inhibitory lectin (OCIL) gene, increases NFATc1 expression to enhance osteoclast differentiation. **B**. Once osteoclasts mature, TIEG1 expression reduces MEK/ERK and AKT/NFκB activation and decreases Bcl2 protein levels, leading to osteoclast apoptosis.

To better understand the mechanistic basis for the defects in osteoclast differentiation and survival, we investigated the role of TIEG1 in activation of pathways known to be involved in these processes. We therefore examined osteoclast precursors for evidence of altered activation of intracellular signaling components downstream of M-CSF and RANKL. We observed that loss of TIEG1 reduced cytokine-mediated activation of NFATc1, the master regulator of osteoclast differentiation [9], [32]. DC-STAMP is required for osteoclast precursor fusion and is stimulated by NFATc1 pathway activation [12]. Loss of TIEG1 also resulted in decreased DC-STAMP expression, confirming that the defect in NFATc1 activation is the probable cause of the defective osteoclast differentiation in TIEG1^−/−^ precursors. We discovered that this defect in osteoclast differentiation is restored by increasing the concentration of RANKL, suggesting that the defect likely resides in RANKL signaling. Our findings therefore suggest that increased TIEG1 expression mediates RANKL-dependent NFATc1 signaling during osteoclast differentiation. To further resolve this, we examined the gene expression levels of crucial signaling pathway components required for osteoclast differentiation including the MCSF receptor, c-fms, the RANKL receptor RANK, and other differentiation-associated genes including TRAFs 2, 3, 5, and 6, OSCAR, TREM2, DAP12, Syk, Fc Receptor γ, MITF, PTEN, and Phospholipase C γ. Of all of these signaling components, RANK was the sole gene whose expression was suppressed in TIEG1^−/−^ osteoclast precursors during the interval in which differentiation was delayed and up-regulated during the time period in which the TIEG1^−/−^ precursors are fusing. The hematopoietic transcription factor PU-1 is required for osteoclast differentiation and function, at least in part, by up-regulating RANK expression [33], [34]. We observed that expression of PU-1 was reduced during the time of differentiation in which TIEG1^−/−^ precursors exhibit a delay in differentiation and expression increased in TIEG1^−/−^ cells as they fuse. RANK signaling leads to increased c-fos expression, which elevates NFATc1 expression and activation to promote osteoclast differentiation [9]. During the time period in which osteoclast differentiation is delayed in same cell line, c-fos gene expression and NFATc-1 protein expression were both decreased in the TIEG1^−/−^ precursors compared to WT cells. This defect was repaired later in differentiation when the TIEG1^−/−^ precursors were fusing. OCIL inhibits osteoclast differentiation by suppressing c-Fos and NFATc1 activation [35], [36]. OCIL levels were elevated in TIEG1^−/−^ precursors during the time of delay and decreased during the period in which the cells were fusing. NFATc1 gene expression was not altered when AdTIEG1 was administered to TIEG1^−/−^ precursors. Since NFATc1 auto-amplifies during osteoclast differentiation, suppression of NFATc1 activation by OCIL may be the cause of the decrease in NFATc1 protein accumulation observed in the TIEG1^−/−^ precursors. This observation supports that expression of NFATc1 in osteoclasts is not directly modulated by TIEG1, but may be due to posttranslational and/or indirect effects such as those of OCIL. The left panel of Figure 8 summarizes our conclusions of the mechanisms by which TIEG1 supports osteoclast precursor fusion leading to multinucleated osteoclasts.

Unexpectedly, MEK/ERK and AKT signaling were elevated in the absence of TIEG1, which is the opposite of what one would expect if these pathways were causing the defect in osteoclast differentiation. We and others have documented that MEK/ERK and AKT pathways are required to support osteoclast survival [5], [27], [30], [37], [38], [39]. We have documented that adenovirus mediated expression of constitutively active AKT or MEK increases survival [5], [40]. These observations support that loss of TIEG1 on the MEK/ERK and AKT pathways are integral to the observed increased survival of osteoclasts. The observed increased activation of MEK/ERK and AKT in spite of reduced osteoclast differentiation is likely to be the mechanism by which loss of TIEG1 expression results in reduced apoptosis (Figure 8, right panel). Having eliminated TIEG1 regulation of gene expression of upstream modulators such as PTEN, consideration must be taken of the known functions of TIEG1 in other bone cells to resolve the mechanism by which TIEG1 promotes osteoclast apoptosis. Our data show that Bcl2 protein levels are higher in TIEG1^−/−^ precursors without detectable changes in mRNA levels. These data support altered translation and/or targeted degradation of Bcl2 protein between the genotypes. The observation that expression of TIEG1 in the TIEG1^−/−^ precursors abrogated Bcl2 protein indicates that this is a cell-autonomous effect of TIEG1 in osteoclast precursors. In addition, it is believed that the apoptosis of osteoclast precursors may be involved in the activation of caspase-9 and that RANKL may promote their survival through Bcl2-induced inhibition of caspase-9 activation [41]. Our findings showed that treatment with RANKL (100 ng) at day 3 significantly increased caspase-9 activation in WT osteoclast precursors when compare to TIEG1^−/−^ precursors (Figure S1A) suggesting that TIEG1 mediates RANKL-induced caspase-9 cleavage in osteoclasts. This observation is confirmed by AdTIEG1 expression in TIEG1^−/−^ precursors (Figure S1B).

Taken together, the findings reported here support that TIEG1 expression in osteoclast precursors accelerates osteoclast differentiation and apoptosis. Rapid turnover of osteoclasts *in vivo* have been observed [42]. This ability of vertebrates to rapidly alter bone resorption allows for fine-tuning of release of calcium from bone. Within the bone environment, osteoclast-mediated release of calcium and osteoblast-mediated incorporation of calcium into bone matrix act in concert to tightly control extracellular calcium levels [43]. Both osteoclasts and osteoblasts respond to local calcium levels by modulation of their respective activities. In osteoclasts, this response leads to decreased resorption [44]. Osteoblast responses to extracellular calcium, in contrast, promote differentiation, survival, and matrix mineralization [45]. Thus, tight control of extracellular calcium may be involved in coupling bone resorption to subsequent bone formation. Rapid modulation of circulating calcium is also a crucial need for organisms since calcium has extensive impacts on most cells. This becomes important in such circumstances as mobilization of calcium during lactation. In breast cells during lactation, low calcium levels in the circulation trigger secretion of Parathyroid Hormone Related Peptide (PTHrP) to increase osteoclast differentiation and bone resorption, which elevates circulating calcium levels for milk production [46]. Once circulating calcium levels are elevated, PTHrP production drops, and the rapid turnover of osteoclasts insures that bone resorption will likewise cease. Our study supports that expression of TIEG1 in osteoclast lineage cells is an integral component of osteoclast turnover. In TIEG1^−/−^ mice, in which TIEG1 is missing in all cells, the defect in osteoblast-mediated support of osteoclast differentiation likely mitigates the cell-autonomous defects in the osteoclast lineage cells that suppress mature osteoclast apoptosis. Testing this hypothesis will require selective deletion of TIEG1 in osteoclast lineage cells, which is a future direction of this project.

## Materials and Methods

### Ethics Statement

Description and characterization of TIEG1^−/−^ mice have been previously described [23], [24]. In this study, 6–12 week old to 16 month old female congenic C57BL/6 WT and TIEG1^−/−^ littermates were used. All animal research was conducted according to guidelines provided by the National Institute of Health and Institute of Laboratory Animal Resources, National Research Council. Mayo Clinic Institutional Animal Care and Use Committee approved all animal studies. Animal protocol (A37708) approved by Mayo Clinic Institutional Animal Care and Use Committee was in accordance with guidelines from the U.S. Public Health Service Policy on Human Care and Use of Laboratory Animals and in compliance with the U.S. Animal Welfare Act.

### Reagents

Recombinant RANKL was expressed in *E. coli* and purified using GST-sepharose columns as described previously [47]. Each batch of recombinant RANKL was tested in dose response studies and the minimal dose that promoted maximal osteoclast differentiation of WT marrow (typically 50 ng/ml) was used for all of the experiments with the exception of the dose-response studies reported here, where the maximum dose tested (100 ng/ml) was twice the dose used for differentiation studies. MCSF was purchased from Research and Diagnostic Systems (Minneapolis, MN). Reagents for tartrate-resistance acid phosphatase (TRAP) staining, Hoechst staining, and other chemicals were purchased from Sigma–Aldrich (St. Louis, MO).

### Osteoclastogenesis assays

Freshly isolated bone marrow cells were used for collection of osteoclast precursors as we have previously reported [47]. Briefly, tibias and femurs were removed from three WT and TIEG1^−/−^ mice for each experiment (6–12 week old to 16 month old) and the metaphyseal ends of the bones were cut and marrow cells were flushed out using a syringe. Marrow cells were cultured in α-MEM plus 10% fetal bovine serum (FBS) containing MCSF (25 ng/ml) for 24 hours. Non-adherent bone marrow cells were collected, seeded at an initial density of 4.5×10^5^ per well in 24-well plates, and cultured in the presence of RANKL (50 ng/ml) and MCSF (25 ng/ml). In parallel, cells were also cultured in the absence of MCSF to verify no mesenchymal cell contamination. Osteoclast precursors were fed with the same media on day 3. To evaluate the effects of RANKL and MCSF on osteoclast differentiation, precursors and mature osteoclasts were fixed in 1% paraformaldehyde and stained with Hoechst 33342 and for TRAP activity as previously described [5].

### Cell proliferation assay

WT and TIEG1^−/−^ non-adherent bone marrow cells were seeded at 1×10^5^ cells/well in 96-well plates and grown at 37°C for 3 h at day 0, 1, 2 and 3 of differentiation. Proliferation/survival was measured on absorbance at 490 nm by using the non-radioactive Cell Titer 96 Aqueous One solution cell proliferation assay (Promega, Madison, WI.) according to the manufacturer's protocol. Averages from eight replicates were compared between WT and TIEG1^−/−^ osteoclasts.

### Colony forming units

To determine the relative numbers of osteoclast precursors in marrow, we analyzed the number of colony forming units Granulocyte-Macrophage (CFU-GM) using MethoCult (Stem Cell Technologies, Vancouver, Canada). One hundred thousand non-adherent bone marrow cells were plated per well in 6 well plates (4 replicates each) for 8 days. Colonies were counted using a phase-contrast microscope.

### RNA extraction and quantitative real time PCR

Total RNA was isolated from the cultured osteoclast precursors and mature osteoclasts using TRIzol reagent (Invitrogen, Carlsbad, CA) according to the manufacturer's instructions. RNA concentration was determined spectroscopically by measuring the absorbance at 260 nm, and RNA purity was assessed by the 260/280 nm absorbance ratio. For quantitative Real Time-Polymerase Chain Reaction (QPCR), first strand cDNA was synthesized from 1 to 2 µg of total RNA using SuperScript II reverse transcriptase following oligo dT priming according to the manufacturer's instructions (Invitrogen, Carlsbad, CA). Custom high-throughput QPCR arrays were utilized to assay 128 genes known to be involved in osteoclastogenesis using the ABI 7900 HT system (Applied Biosystem, Foster City, CA). All genes were compared between WT and TIEG^−/−^ osteoclast precursors and mature osteoclasts. Up- and down-regulated genes in TIEG1^−/−^ osteoclast precursors (n = 3) relative to those obtained from WT mice (n = 3) were confirmed individually using a Bio-Rad Q-PCR (iCycler, Hercules, CA). Sequences of primer pairs used in these QPCR experiments are listed in Table S3. Primer pairs were designed to span introns in order to prevent potential amplification of any contaminating genomic DNA. Gene expression levels were calibrated using endogenous TBP expression levels. The differences between the mean Ct values of genes were denoted (Δ-Ct) and the difference between Δ -Ct values of test genes and the Δ-Ct value of TBP was calculated as ΔΔ -Ct. The log_2_(ΔΔ -Ct) was used to determine the relative quantification value of expression and values were compared between WT and TIEG1^−/−^ cells.

### Western blot analysis and antibodies

Non-adherent bone marrow cells were cultured as described above and subsequently lysed as previously reported [47]. Protein concentrations were determined using the BCA protein assay kit (Pierce, Rockford, IL). Proteins were separated using 12% SDS-PAGE gels followed by electroblotting to Immobilon-P membranes (Millipore, Bedford, MA) using a transfer buffer containing 50 mM Tris, 40 mM glycine, 0.1% SDS, and 20% methanol at pH 9.2. Membranes were blocked by incubation in 1× PBS containing 5% fat-free dry milk for 1 h at room temperature. Blots were incubated with the following primary antibodies: AKT, pAKT, MEK1/2, pMEK1/2, ERK, pERK1/2, Caspase-9 (Cell Signaling Technology, Boston, MA), NFATc1 (BD Pharmingen, San Jose, CA), pNFATc1, Cathepsin K, Bcl-2 (Santa Cruz Biotechnology, Santa Cruz, CA), and β-actin (Sigma–Aldrich). Incubation with primary antibodies was carried out overnight at 4°C in a dilution buffer containing 1× PBS, 0.1% tween-20 and 5% fat-free dry milk. Blots were subsequently probed with horseradish peroxidase-conjugated anti-mouse or anti-rabbit secondary antibodies (Amersham Biosciences, Buckinghamshire, England) diluted 1∶5000 in 1× PBS, 0.1% tween-20 and 5% fat-free dry milk. Signals were visualized using the ECL Plus detection system (Amersham Biosciences) according to the manufacturer's instructions.

### Adenoviral infections

Mouse TIEG1 adenovirus was generated under contract by Vector BioLabs (Philadelphia, PA). TIEG1^−/−^ osteoclast precursors were infected at day 2 with either adenovirus expressing TIEG1 or vector alone and TIEG1 expression was monitored by QPCR at day 3 and 4 to determine relative TIEG1 expression levels. To determine optimum infection conditions, TIEG1^−/−^ osteoclast precursors were infected at a range of multiplicity of infections (0 to 50 MOI) and the MOI resulting is expression of TIEG1 at levels similar to those observed in WT cells was selected for use in all studies (25 MOI). The infected cells were differentiated as described above and subsequently fixed and stained with Hoechst and for TRAP activity. For protein and RNA analysis, infected cells were washed twice with 1× PBS and RNA and proteins were harvested as described above.

### siRNA inhibition of TIEG1 expression in wildtype bone marrow cells

Targeted interfering RNA (Accell siRNA) for TIEG1, r(AAUGGAACUAAUUUCUGAA)d(TT)), was custom designed by Darmacon (Lafayette, CO). siRNA duplexes were transiently transfected into WT osteoclast precursors using HiPerfect transfection reagent from QIAGEN according to the manufacturer's instructions. Transient transfection of 100 mM siTIEG1 oligonucleotides was performed in OPTI-MEM media at day 2 and the efficiency of TIEG1 knock-down was confirmed by QPCR as described above. On day 3, cells were fed with α-MEM plus 10% FBS containing RANKL (50 ng/ml) and MCSF (25 ng/ml). To evaluate the effects of siTIEG1 transfection on osteoclast differentiation, cells were fixed in 1% paraformaldehyde and stained with Hoechst and for TRAP activity as described previously.

### Confocal Microscopy

The localization of phospho and total NFATc1 in cultured WT and TIEG1^−/−^ precursors were examined by immuno florescence histochemistry using a confocal microscope. Cells were fixed in 1% paraformaldehyde for at least 30 min and washed twice with 1× PBS. Cells were permeabilized with 0.2% Triton-X in PBS for 30 min and incubated for an additional 30 min in heat-inactivated 5% FBS to block non-specific binding and subsequently incubated with 2 ug/mL rabbit polyclonal phospho-NFATc1 or NFATc1 antibodies in PBS for an additional 60 min. Cells were washed twice with PBS and stained with Texas Red- or FITC-conjugated secondary IgG antibodies (Santa Cruz Biotechnology, Santa Cruz, CA) for 60 minutes. DAPI was used for counter-staining of nuclei.

### Statistics

All data are presented as mean +/− SD. Statistical significance was determined by 2-tailed Student's T-test using Microsoft Excel software.

## Supporting Information

Figure S1 **A**. Dose-response of RANKL signaling effects on caspase 9 expression in osteoclast precursors at day 3. The precursor cells were treated with the indicated concentrations of RANKL in the presence of MCSF. **B**. AdTIEG1 expression effects on caspase 9 expression in osteoclast precursors. Osteoclast precursors from TIEG1^−/−^ mice were cultured and infected at Day 2 with vector (AdVec) or TIEG1 adenovirus (AdTIEG) at an MOI of 25.
(TIF)

Table S1 **Genes associated with osteoclast fusion.** Relative expression levels are reported as mean ± standard deviation (SD). *The fold changes are the ratios of the mean relative expression levels in WT over the mean relative expression levels in KO osteoclasts (n = 3). ^a^ *p*<0.05.
(TIF)

Table S2 **Genes not altered with the loss of TIEG1 were associated with osteoclast fusion and Bcl2 family.** Relative expression levels are reported as mean ± standard deviation (SD). *The fold changes are the ratios of the mean relative expression levels in WT over the mean relative expression levels in KO osteoclasts (n = 3).
(TIF)

Table S3 **Oligonucleotide primer pairs (Sense and Antisense) used for Q-PCR.**
(TIF)
