# Supplementary figures and images for: TGF-β Inducible Early Gene 1 Regulates Osteoclast Differentiation and Survival by Mediating the NFATc1, AKT, and MEK/ERK Signaling Pathways
Source: PLoS One. 2011 Mar 14;6(3):e17522. doi: 10.1371/journal.pone.0017522 (PMC3056664; doi:10.1371/journal.pone.0017522)

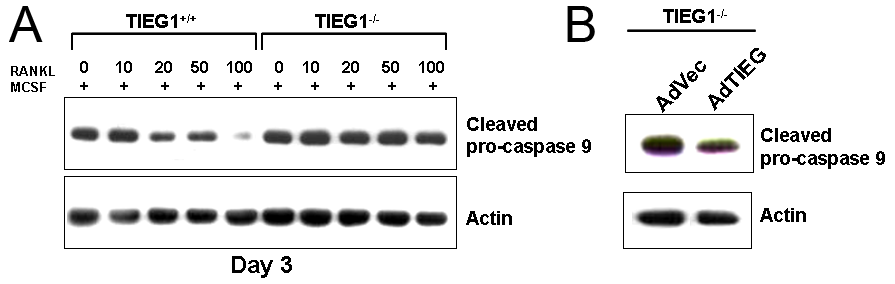

Supplement: Figure S1 — A. Dose-response of RANKL signaling effects on caspase 9 expression in osteoclast precursors at day 3. The precursor cells were treated with the indicated concentrations of RANKL in the presence of MCSF. B. AdTIEG1 expression effects on caspase 9 expression in osteoclast precursors. Osteoclast precursors from TIEG1−/− mice were cultured and infected at Day 2 with vector (AdVec) or TIEG1 adenovirus (AdTIEG) at an MOI of 25. (TIF) [file pone.0017522.s001.tif]

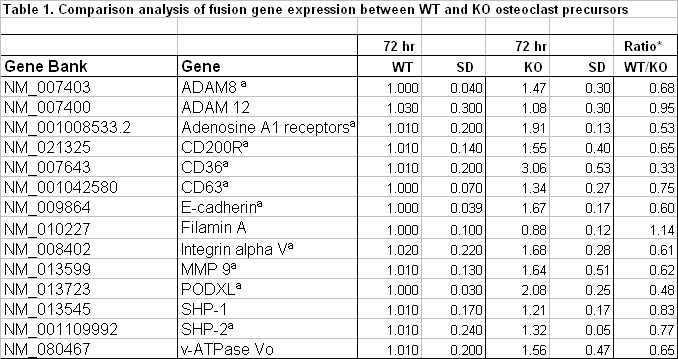

Supplement: Table S1 — Genes associated with osteoclast fusion. Relative expression levels are reported as mean ± standard deviation (SD). *The fold changes are the ratios of the mean relative expression levels in WT over the mean relative expression levels in KO osteoclasts (n = 3). a p<0.05. (TIF) [file pone.0017522.s002.tif]

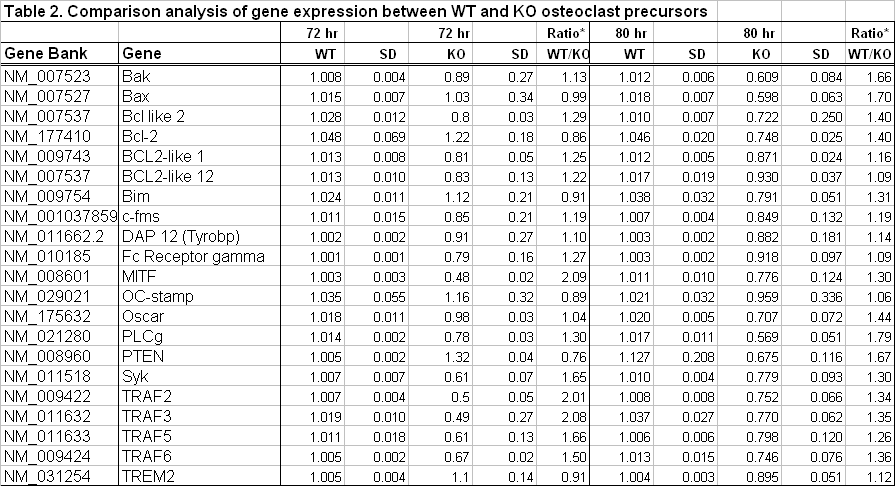

Supplement: Table S2 — Genes not altered with the loss of TIEG1 were associated with osteoclast fusion and Bcl2 family. Relative expression levels are reported as mean ± standard deviation (SD). *The fold changes are the ratios of the mean relative expression levels in WT over the mean relative expression levels in KO osteoclasts (n = 3). (TIF) [file pone.0017522.s003.tif]

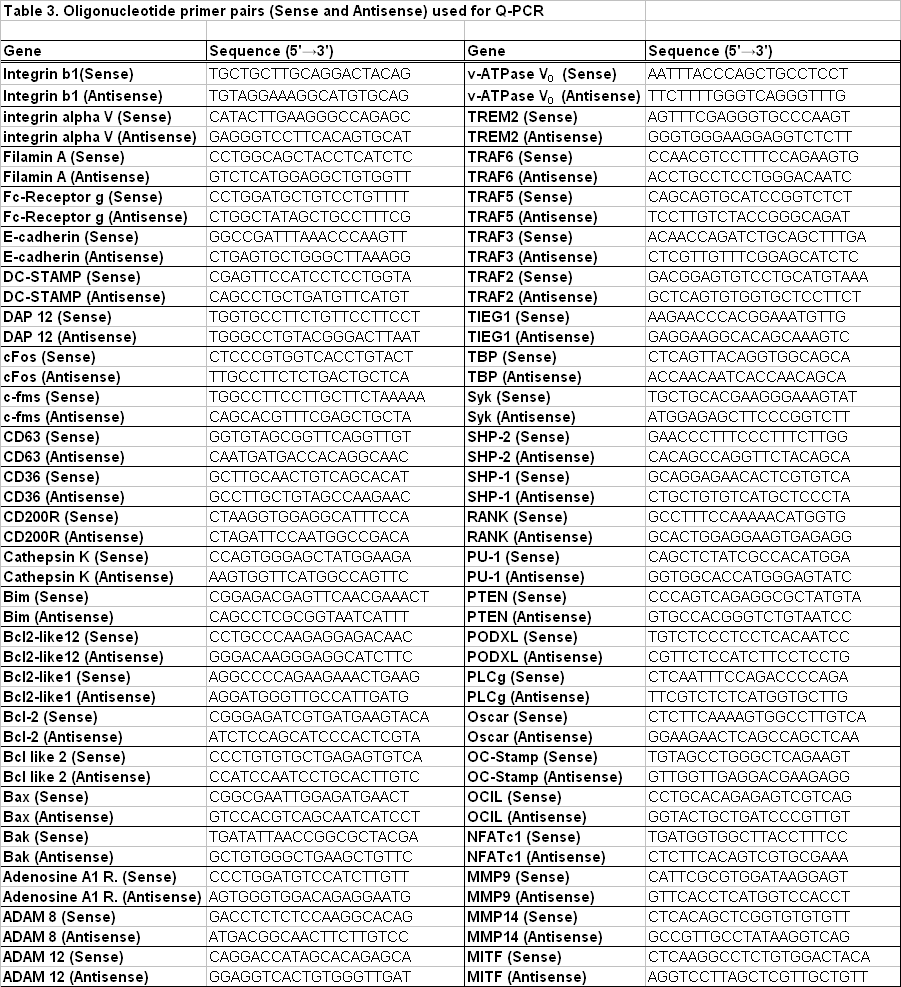

Supplement: Table S3 — Oligonucleotide primer pairs (Sense and Antisense) used for Q-PCR. (TIF) [file pone.0017522.s004.tif]
